# The tide has turned: incidence of depression declined in community living young-old adults over one decade

**DOI:** 10.1017/S2045796018000811

**Published:** 2019-01-26

**Authors:** H.W. Jeuring, E.O. Hoogendijk, H.C. Comijs, D.J.H. Deeg, A.T.F. Beekman, M. Huisman, M.L. Stek

**Affiliations:** 1 Department of Psychiatry, GGZ inGeest/VU University Medical Center, Amsterdam, The Netherlands; 2 Department of Epidemiology and Biostatistics and the Amsterdam Public Health Research Institute, VU University Medical Center, Amsterdam, The Netherlands; 3 Department of Sociology, VU University, Amsterdam, The Netherlands

**Keywords:** Depression, epidemiology, incidence, risk factors

## Abstract

**Aims:**

Studying birth-cohort differences in depression incidence and their explanatory factors may provide insight into the aetiology of depression and could help to optimise prevention strategies to reduce the worldwide burden of depression.

**Methods:**

Data were used from the Longitudinal Aging Study Amsterdam, a nationally representative study among community dwelling older adults in the Netherlands. Cohort differences in depression incidence over a 10-year-period (score ⩾16 on the Center for Epidemiologic Studies Depression scale) were tested using a cohort-sequential-longitudinal-design, comparing two identically measured cohorts of non-depressed 55–64-year-olds, born 10-years apart. Baseline measurements took place in 1992/93 (early cohort, *n* = 794), and 2002/03 (recent cohort, *n* = 771). As indicated by the dynamic equilibrium model of depression, potential explanatory factors were distinguished in risk and protective factors.

**Results:**

The incidence rates for depression in the early and recent cohort were 1.91 (95% confidence interval (CI) 1.59–2.27) and 1.60 (95% CI 1.31–1.94) per 100 person-years, respectively. A 29% risk reduction in depression incidence was observed in the recent cohort (HR_cohort_: 0.71, 95% CI 0.54–0.92, *p* = 0.011), as compared with the early cohort, even though average levels of risk factors such as chronic disease and functional limitations had increased. This reduction was primarily explained by increased levels of education, mastery and labour market participation.

**Conclusions:**

These findings suggest that favourable developments of protective factors have counterbalanced unfavourable effects of risk factors on the incidence of depression, resulting in a net reduction of depression incidence among young-old adults. However, maintaining a good physical health must be a priority to further decrease depression rates.

## Introduction

It needs no introduction that depression is a major contributor to the global burden of disease at all ages (Ferrari *et al*., 2013). Although there has been an increasing awareness in the recent decades of the need to better recognise and treat depression (Kohls *et al*., 2017), a majority of studies have observed an increase in depression rates among recent generations (Wittchen and Uhmann, 2010). These findings have been largely based on prevalence studies (e.g. Compton *et al*., 2006; Jeuring *et al*., 2018; Weinberger *et al*., 2018), while only very few have been able to investigate cohort differences in the incidence of depression (Waraich *et al*., 2004). Whereas prevalence rates provide information about the population burden of depression, they are the net-result of the combination of the incidence and the course of depression. For studying putative changes in the occurrence of new episodes of depression and the factors that are responsible for these trends, incidence rates are more accurate. Information on trends in incidence rates and their underlying mechanisms is essential for the design of prevention strategies to help to reduce the worldwide burden of depression.

Reasons for the lack of knowledge on cohort differences in the incidence of depression may be that the required nationwide prospective studies are scarce, expensive and time-consuming. Moreover, available studies provided evidence for both increasing and decreasing trends in incidence of depression, implying that the incidence fluctuates with time and place, and that studies should be repeated in different contexts. The Lundby study in Sweden has found a twofold increase in the incidence rate of depression when a 10-year period, 1947–1957, was compared with a subsequent 15-year period, 1957–1972 (Hagnell *et al*., 1982). However, a slight decrease in the incidence rate within the Lundby study had been observed in the period 1972–1997, as compared with the period 1947–1972 (Mattisson *et al*., 2005). The Sterling County Study in Canada has demonstrated a stable annual incidence rate of depression for the period 1952–1992 (Murphy *et al*., 2000). A study on data collected from general practitioners in the UK from 1996 to 2006 has demonstrated a decrease in the incidence of depression diagnoses, but an increase in the registration of depressive symptoms (Rait *et al*., 2009). Most of these studies lacked the ability to identify explanatory factors for the trends found in depression incidence. Studying explanatory factors may reveal underlying mechanisms of trends in depression incidence, which can subsequently be addressed in preventive policy and medicine.

A useful model to understand the aetiology and onset of depression is the dynamic equilibrium model of risk and protective factors, in which the balance of risk factors relative to protective factors determines the observed changes in incidence (Fiske *et al*., 2009). In a large meta-analysis on risk factors of incident depression among community dwelling older adults (aged ⩾50 years), Cole (2003) identified new medical illness, poor health status, prior depression, poor self-perceived-health, bereavement, sleep disturbance, disability and female sex as the most important risk factors (Cole and Dendukuri, 2003). These findings suggest that health-related problems may be a central realm of risk of incident depression among middle-aged and older adults. In a previous study on cohort differences in the prevalence of depression, we confirmed that an increase in health problems was associated with an increase in depression prevalence in more recent cohorts of 55–64-year-olds in comparison with previous cohorts of age-peers (Jeuring *et al*., 2018). Simultaneously, we found that average increases in protective factors, such as educational level, labour market participation and sense of mastery, prevented the depression prevalence rates from having increased even further. These findings emphasise the importance of incorporating protective factors in studies on the incidence of depression (Jeuring *et al*., 2018). From these findings, it may be assumed that the shift in depression prevalence among 55–64-year-olds is the net result of two broad underlying trends in recent generations, with opposing effects on depression: the shift towards increased levels of physical health problems (increased risk) on the one hand, and an improvement in overall socio-economic resources, such as education, access to work and mastery (increased protection) on the other. Whether these trends in risk and protective factors of depression prevalence also explain cohort differences in depression incidence has not been studied yet.

The Longitudinal Aging Study Amsterdam (LASA) allows the identification and explanation of birth-cohort differences in the incidence of depression by using a long-term (10 years) follow-up in two identically measured nationally representative samples of 55–64-year-olds, who were non-depressed at baseline (Huisman *et al*., 2011; Hoogendijk *et al*., 2016). From a clinical point of view, 55–64-year-olds are a suitable target for prevention purposes of late-life depression. Moreover, this age group is of interest because they are young enough to have experienced changes in psychosocial and socioeconomic circumstances, and old enough to have experienced changes in health status, such as physical illness and functional limitations (Jeuring *et al*., 2018).

The aim of the current study is to investigate and explain cohort differences in the incidence of depression. Based on our previous finding of an increase in major depression prevalence (Jeuring *et al*., 2018), we expected to find a higher incidence of depression in the recent cohort compared with the early cohort and that this increase in incidence rate could be explained by higher average levels of health-related risk factors in the recent cohort, as compared with the earlier cohort.

## Methods

### Study sample

Data were used from the LASA, an ongoing prospective cohort-sequential study among older adults in the Netherlands. Sampling procedures have been described previously (Huisman *et al*., 2011; Hoogendijk *et al*., 2016). In short, in 1992/93 the first cohort (*N* = 3107, birth years 1908–1937) was recruited from the population registries of 11 municipalities in three geographic areas of the Netherlands including a random sample of 55–84-year old men and women, stratified by age and sex according to the expected 5-year mortality. The cooperation rate of the first cohort was 62%, also for the 55–64-year-olds subsample (birth years: 1928–1937). Follow-ups were conducted in 1995/96 (*N* = 2545), 1998/99 (*N* = 2076) and 2001/02 (*N* = 1691). Exactly 10 years after the first cohort, a new cohort (*N* = 1,002, birth years 1938–1947) was recruited in 2002/03 including a random sample of 55–64-year-olds selected from the same sampling frame and measured identically to the first cohort. The cooperation rate for the second cohort was 62%. In subsequent observational cycles, respondents from the second cohort were combined with those from the first cohort. Follow-ups were conducted in 2005/06 (*N* = 1257), 2008/09 (*N* = 985) and 2011/12 (*N* = 614). All interviews were conducted in the homes of the respondents by trained interviewers. Written informed consent was obtained from all respondents. The Ethical Review Board of the VU University Medical Center approved the study.

To test our hypothesis on cohort differences in the incidence of depression, information was used on these two cohorts, for which follow-up of 10 years was available at the time of this study. From both cohorts, respondents with a strict age limit of 55–64-years at baseline were included. Four observation cycles were used for each cohort. At the fourth observational cycle (2001–02 and 2011–12, respectively) respondents were aged 65–74. Respondents were excluded when having clinically relevant depression at baseline or lacked at least one follow-up measurement. The final sample consisted of *N* = 1565 respondents and a total of 12 695 person-year observations. Of these 1565 respondents, 794 respondents were from the first cohort and 771 respondents from the second cohort.

## Measures

### Dependent variable

The Center for Epidemiologic Studies Depression scale (CES-D) was applied to identify respondents with clinically relevant depression at each observational cycle (Radloff, 1977). Clinically relevant depression was defined by a CES-D score ⩾16. The cut-off of 16 is previously validated in LASA, and used in previous studies on depression epidemiology. The psychometric properties of the CES-D were found to be good (Beekman *et al*., 1997). Incident depression was defined as onset of a new depression (CES-D ⩾ 16) during the observation period. Respondents without clinically relevant depression (CES-D < 16) were indicated as having no depression. Respondents with previous episodes of depression were not excluded due to unavailability of this information for all cohort members.

### Main independent variable

A dichotomous variable denoting birth-cohort was constructed, with values for belonging to the ‘early cohort’ with baseline measurement in 1992/93, and the ‘recent cohort’ with baseline measurement in 2002/03.

### Explanatory independent variables

Based on two literature reviews on risk factors of incident depression among community dwelling older adults aged 55 years or older (Cole and Dendukuri, 2003; Vink *et al*., 2008), putative risk and protective factors were included from biological, psychological and social domains of functioning. According to the literature and based on biological plausibility, factors were considered either as risk or protective factors.

The following risk factors were included. The *number of chronic diseases* was assessed by self-report on current diseases and included cardiovascular disease, diabetes mellitus, cancer, cerebrovascular accident, arthritis and chronic non-specific pulmonary disease (range, 0–7) (Kriegsman *et al*., 1996). *Functional limitations* were measured by self-report and dichotomised in ‘none’ *v*. ‘one or more’ limitations (McWhinnie, 1981). *Body mass index* was calculated as measured body weight (kg) divided by measured height (m^2^). *Pain* was measured with the Nottingham Pain Profile scale (range, 5–10) (Landy *et al*., 1991). *Sleep problems* were measured with a four-item self-completion questionnaire (range, 3–12) (Landy *et al*., 1991). *Alcohol consumption* was measured by the number of alcohol units consumed per day (u/d) and categorised into: abstainer (0 u/d), moderate (men, 1–3 u/d; women, 1–2 u/d) and excessive (men, ⩾4 u/d; women, ⩾3 u/d) (Netherlands Central Bureau of Statistics, 1989). *Smoking* was dichotomised into ‘current smoker or stopped ⩽15 years ago’ *v*. ‘never smoked or stopped >15 years’ (Visser *et al*., 1999). *Physical activity* was measured by the LASA Physical Activity Questionnaire, from which the total time in min per day spent on physical activity was calculated (Stel *et al*., 2004). *Neuroticism* was measured with a 25-items subscale from the 36-item Dutch Personality Questionnaire (range, 0–50) (Luteijn *et al*., 1975). *Loneliness* was assessed with the De Jong-Gierveld Loneliness Scale (range, 0–11) (de Jong-Gierveld and Kamphuls, 1985).

The following protective factors were included. *Religiousness* was dichotomised in having a religion or not. *Partner status* was dichotomised in having a partner in or outside the household *v*. having no partner. *Education* was based on the number of years of education followed (range, 5–18). *Labour market participation* was assessed by self-report of having a paid job for more than 1 h per week. *Physical performance* was measured with three performance tests, including walking, chair stand and balance (range, 0–12, with higher scores indicating better performance) (Penninx *et al*., 2000). *Mastery* was measured with a translated and abbreviated version of the Pearlin Mastery Scale (range, 5–25) (Pearlin and Schooler, 1978). *Personal network size* was based on the total number of network members the respondent had regular contact with (range, 0–75); and the *exchange of social support* (both instrumental and emotional) was collected for nine network members whom the respondent had the most frequent contact with (range, 0–36) (Van Tilburg, 1998).

Use of *antidepressants* and *benzodiazepines* were assessed by directly recording the medication from drug containers in the home of the respondents (Sonnenberg *et al*., 2008). All measurement instruments were either previously validated in comparable samples in the Netherlands or in LASA pilot studies (Deeg *et al*., 1993). Because the dataset contained minimal 5% and maximal 25% missing values in some risk and protective factors, multiple imputation was performed, including 25 imputations and 50 iterations.

### Statistical analyses

Descriptive statistics were performed on complete-cases data of the two cohorts pooled. The early cohort's data were weighted according to the distribution of age and sex in the recent cohort. This was done to make sure that changes in the incidence of depression, risk and protective factors reflected cohort differences and was not due to distributional differences in age and sex. *χ*^2^, *t* tests and Kruskal–Wallis tests were performed to examine cohort differences in risk and protective factors of depression.

Cox proportional hazard regression models the incidence or hazard rate, the number of new cases of depression per population at-risk per unit time. Respondents who developed incident depression at follow-up were labelled as ‘cases’, in which the exact date of the CES-D interview was used as survival time. Those that did not develop depression, or dropped-out (death or loss to follow-up) were all treated as ‘censored’. Further analyses performed with Cox proportional hazard regression were not weighted since all models were standard adjusted for age and sex. A basic model was created to test the association between ‘cohort’ and ‘depression’, adjusted for age and sex, to estimate the presence and degree of a cohort difference in the incidence of depression. The recent cohort was compared with the early cohort (=reference). All risk and protective factors were separately investigated for their explanatory ability. Potential explanatory factors were manually entered one by one into the basic model and the % change in hazards ratio of ‘cohort’ (HR_cohort_) was estimated (Table 2). The % change in (HR_cohort_) was calculated with following formulas: ((HR_basic model_ − HR_model *x*_)/(HR_basic model_)) × 100 (Richter *et al*., 2012).

Factors were considered to be potentially explanatory when the magnitude of the association of cohort with depression incidence (HR_cohort_) was reduced after adding them to the regression model: thus decrease in HR if HR > 1 or increase in HR if HR < 1. Explanatory factors were considered to be suppressors when the opposite was observed: the magnitude of the association (HR_cohort_) became stronger after adding them to the regression model: thus decrease in HR if HR < 1 or increase in HR if HR > 1. It is important to take into account suppressors in an explanatory analysis like this, because they indicate how much stronger the association (HR_cohort_) would have been if these suppressing factors had remained stable over time.

Finally, multivariable analyses were performed to estimate the total percentage that could be explained by adjusting the basic model subsequently for the overall influence of suppressors, the overall influence of explanatory risk factors and finally for the overall influence of explanatory protective factors (Table 3). Data analyses were conducted with SPSS v22.

## Results

### Cohort difference in depression incidence

**Fig. 1.**
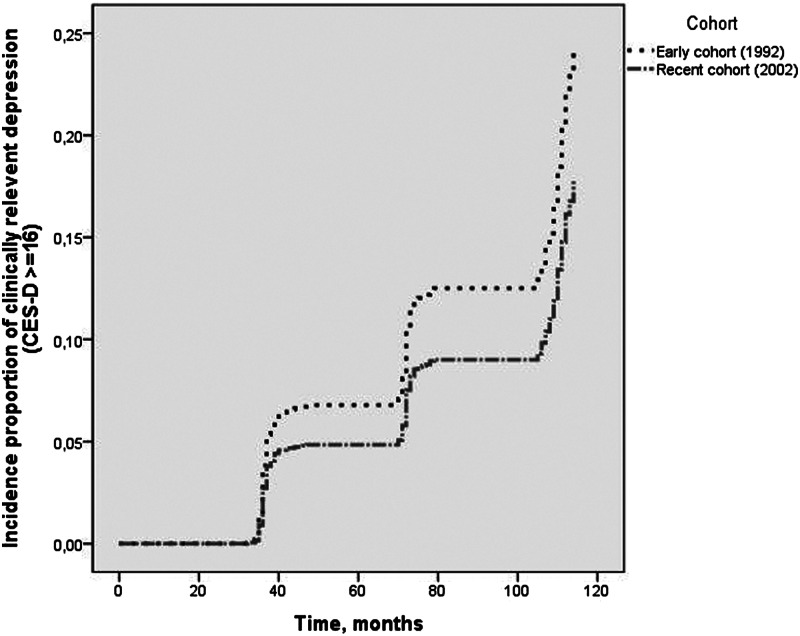
Decline in depression incidence among young-old adults between 2002 and 2012 *v*. 1992 and 2002. The figure is an output of SPSS, delivered by a Cox proportional hazards regression, adjusted for sex and age. On the *Y*-axis of the figure, the cumulative incidence proportion of depression (CES-D ⩾ 16) for both cohorts is shown. On the *X*-axis, the survival time in months is shown (i.e. onset time to incident depression).

From the 794 non-depressed respondents in the early cohort at baseline, 122 (15.4%) developed incident depression at follow-up. From 771 non-depressed respondents in the recent cohort at baseline, 101 (13.1%) developed incident depression at follow-up. The total time at risk in the early cohort was 76 785 months (6399 years), whereas the total time at risk in the recent cohort was 75 559 months (6297 years). The incidence rates for the early and recent cohort were 1.91 (95% CI 1.59–2.27) and 1.60 (95% CI 1.31–1.94) per 100 person-years, respectively. The risk of developing incident depression in the recent cohort, adjusted for sex and age, was 29% less as compared with the early cohort (HR: 0.71, 95% CI 0.54–0.92), which is illustrated in Fig. 1.

### Cohort differences in risk and protective factors

Table 1 shows the cohort differences in risk and protective factors of depression between the early (1992/93) and recent (2002/03) cohort. The recent cohort had on average a higher level of education, more labour market participation, higher levels of mastery, of physical performance, of given instrumental and of given emotional support than the previous cohort. It also had lower average levels of neuroticism and smoking, but more chronic diseases, and functional limitations, a higher average body mass index, lower levels of physical activity and more excessive alcohol use, compared with the previous cohort. Finally, the use of antidepressants was higher in the recent cohort.

**Table 1.** Baseline characteristics of non-depressed respondents by cohort

Variables	Early cohort 1992/93	Recent cohort 2002/03	*p* Value
No. observations, unweighted	794	771	
Female, no. (%)	406 (51.1)	385 (49.9)	0.64
Age, 55–64, mean (s.d.), years	60.1 (2.8)	59.9 (2.9)	0.084
No. observations, weighted	793	771	
Protective factors			
Education, mean (s.d.), years	9.6 (3.3)	10.6 (3.4)	**<0.001**
Labour market participation, no. (%), yes	251 (32.0)	358 (46.4)	**<0.001**
Mastery, mean (s.d.)	18.3 (3.1)	18.8 (3.0)	**0.003**
Religious, no. (%), yes	482 (60.7)	413 (53.6)	**0.004**
Partner, no. (%), yes	678 (85.4)	674 (87.4)	0.24
Network size, median (IQR)	14.0 (12.0)	14.0 (12.0)	0.68
Exchange of social support, mean (s.d.)			
Instrumental support given	16.1 (7.0)	17.4 (6.9)	**<0.001**
Instrumental support received	14.5 (6.4)	14.8 (6.3)	0.36
Emotional support given	21.6 (7.8)	24.2 (7.4)	**<0.001**
Emotional support received	23.1 (7.5)	22.6 (7.6)	0.24
Physical performance, mean (s.d.)	8.7 (2.3)	9.2 (2.2)	**<0.001**
Risk factors			
Neuroticism, median (IQR)	4.0 (6.0)	4.0 (6.0)	**0.009**
Loneliness, median (IQR)	0.0 (2.0)	1.0 (2.0)	0.80
Sleep problems, mean (s.d.)	5.4 (2.0)	5.5 (2.0)	0.66
Pain, median (IQR)	5.0 (0.0)	5.0 (1.0)	0.11
⩾1 Chronic diseases, no. (%)	359 (45.3)	396 (51.4)	**0.016**
⩾1 Functional limitations, no. (%)	117 (14.8)	164 (21.3)	**0.001**
Body mass index, median (IQR)	26.4 (4.3)	27.0 (5.1)	**0.003**
Physical activity, median (IQR), min/day	173.6 (158.7)	143.2 (137.5)	**<0.001**
Alcohol use			**<0.001**
Abstainer	101 (13.9)	48 (6.6)	
Moderate use	543 (74.7)	534 (73.2)	
Excessive use	83 (11.4)	148 (20.3)	
Smoking, no. (%)	366 (50.1)	314 (43.0)	**0.006**
CES-D score at baseline, mean (s.d.)	4.9 (4.1)	5.4 (4.2)	**0.021**
Incident depression (CES-D ⩾ 16) at follow-up, no. (%)	122 (15.4)	101 (13.1)	0.20
Psychotropic medication			
Antidepressant use, no. (%), yes	5 (0.7)	22 (3.0)	**0.001**
Benzodiazepine use, no. (%), yes	42 (5.7)	39 (5.3)	0.74

No., number; s.d., standard deviation; IQR, interquartile range; CES-D, Center for Epidemiologic Studies Depression scale.

Bold values are statistically significant at *p* < 0.05. *χ*^2^ values have been computed for categorical variables, *t*-values for interval variables and independent-sample Kruskal–Wallis tests were conducted to determine non-parametric variables.

### Explaining cohort difference in depression incidence

The contribution of each risk and protective factor to the difference in depression incidence between the cohorts is shown in Table 2. The differences in education (−9.9%), mastery (−7.0%) and neuroticism (−7.0%) contributed most to the difference in depression incidence. On the other hand, the high levels of functional limitations (+9.9%), chronic diseases (+5.6%), and excessive alcohol use (+4.2%) in the recent cohort suppressed the association of cohort with depression incidence. In other words, had these health-related factors remained constant, the incidence of depression would have decreased even further. Table 3 shows that the risk of developing depression in the recent cohort was 42% less (HR: 0.58, 95% CI: 0.43–0.79) as compared with the early cohort, after adjustment for all suppressors (model V).

**Table 2.** Factors associated with the decrease in the incidence of depression in the recent cohort

	Incidence of depression (CES-D ⩾ 16)			
	HR_cohort_	HR_change_ (%)	95% CI	*p* Value
Recent cohort (*v*. early), unadjusted	0.70		0.54–0.92	0.010
Recent cohort (*v*. early), age and sex-adjusted	0.71	Reference	0.54–0.92	0.011
Protective factors				
Education	0.78	−9.9	0.59–1.02	0.069
Labour market participation	0.75	−5.6	0.65–0.86	0.032
Mastery	0.76	−7.0	0.58–0.99	0.049
Religious	0.71	0	0.54–0.93	0.011
Partner	0.71	0	0.54–0.93	0.013
Network size	0.72	−1.4	0.67–0.88	0.054
Exchange of social support				
Instrumental support given	0.74	−4.2	0.57–0.96	0.027
Instrumental support received	0.71	0	0.62–0.81	0.011
Emotional support given	0.74	−4.2	0.56–0.97	0.028
Emotional support received	0.71	0	0.62–0.81	0.011
Physical performance	0.73	−2.8	0.56–0.96	0.023
Risk factors				
Neuroticism	0.76	−7.0	0.58–0.99	0.046
Loneliness	0.71	0	0.62–0.81	0.012
Sleep problems	0.70^a^	+1.4	0.53–0.93	0.015
Pain	0.69^a^	+2.8	0.53–0.90	0.007
⩾One chronic disease	0.67^a^	+5.6	0.51–0.88	0.004
⩾One functional limitation	0.64^a^	+9.9	0.49–0.84	0.001
Body mass index	0.69^a^	+2.8	0.53–0.91	0.008
Physical activity	0.70^a^	+1.4	0.53–0.91	0.009
Alcohol use	0.68^a^	+4.2	0.52–0.90	0.007
Smoking	0.72	−1.4	0.55–0.95	0.018
Psychotropic medication				
Antidepressant use	0.71	0	0.54–0.93	0.013
Benzodiazepine use	0.71	0	0.54–0.93	0.013

HR, hazard ratio; CES-D, Center for Epidemiologic Studies Depression scale.

a Suppressors.

**Table 3.** Multivariate models explaining the decrease in depression incidence in the recent cohort

	Incidence of depression (CES-D ⩾ 16)			
	HR_cohort_	HR_change_ (%)	95% CI	*p* Value
Blockwise				
Basic Model (BM)	0.71	Reference	0.54–0.92	0.01
Model II. BM + increase in protective factors	0.93	−31	0.70–1.24	0.62
Model III. BM + decrease in risk factors	0.77	−9	0.59–1.01	0.06
Model IV. BM + increase in risk factors (suppressors)	0.58	+18	0.43–0.79	<0.001
Stepwise				
Model V. Model IV (suppressors)	0.58	Reference	0.43–0.79	<0.001
Model VI. Model V + III (explanatory risk factors)	0.64	−10	0.47–0.86	0.003
Model VII. Model VI + II (explanatory protective factors)	0.79	−36	0.57–1.09	0.15

CES-D: Center for Epidemiologic Studies Depression scale. Variables included in models: basic model (BM) = cohort, adjusted for age and sex; model II = BM + education, mastery, labour market participation, network size, instrumental and emotional support given, physical performance; model III = BM +  neuroticism and smoking; model IV = BM + functional limitations, chronic diseases, alcohol use, pain, body mass index, sleep problems, physical activity.

The decrease in risk factors (neuroticism and smoking) explained 10% of the reduction in depression incidence (model VI), but the overall increase in protective factors (education, mastery, labour market participation, network size, instrumental and emotional support given, physical performance) explained the most part of the reduction in depression incidence (model VII). In total, 36% of the difference in depression incidence between the cohorts could be explained by all these risk and protective factors together.

## Discussion

This study found a substantial cohort difference in the 10-year incidence of depression between two cohorts of 55–64-year-olds: those from the recent cohort developed incident depression less often than the earlier cohort. The difference amounted to a 29% lower incidence risk observed in the recent cohort. The difference was primarily related to favourable developments in protective and resilience related factors, including an increase in levels of education, mastery and labour market participation. Had risk factors, such as chronic diseases and functional limitations, not increased in the recent cohort compared with the earlier cohort, the incidence difference would have been even larger. Although this finding indicates that chronic diseases and functional limitations are indeed important risk factors for developing depression, their relatively high levels in the recent cohort had not resulted in an increase in depression incidence between the cohorts, thereby falsifying our initial hypothesis. In terms of the dynamic equilibrium theory of depression, we conclude that the favourable developments in protective factors have outweighed the concurrent negative effects of developments in risk factors of depression.

Our study adds new knowledge to previous work, because published epidemiological studies on trends in depression rates have mainly focused on cohort differences in the prevalence (Waraich *et al*., 2004), rather than incidence of depression. The majority of these prevalence studies have reported an increase in depression rates (Wittchen and Uhmann, 2010). The observed incidence rates of depression from the early (1.9 per 100 person-years) and recent cohort (1.6 per 100 person-years) are both of comparable magnitude as those in other studies (Waraich *et al*., 2004). However, increasing (Hagnell *et al*., 1982), decreasing (Eaton *et al*., 2007; Rait *et al*., 2009) and stable trends in depression incidence have all been reported (Murphy *et al*., 2000). Comparing these studies is extremely difficult, because different samples and study designs have been used, and findings refer to different period and social contexts. When comparing our previous trend study in depression prevalence (higher rates) (Jeuring *et al*., 2018), with the current trend study in depression incidence (lower rates), it may be suggested that a higher prevalence is due to an increasing chronicity of depression in more recent cohorts, which has also been suggested by Eaton and colleagues using data from the Baltimore Epidemiologic Catchment Area Study (Eaton *et al*., 2007).

To our knowledge no previous study has addressed explanatory factors of cohort differences in a systematic way by using the dynamic equilibrium theory of depression. Our study identified a combination of risk and protective factors that together explained approximately one third of the difference in depression incidence. The protective role of education and sense of mastery in preventing the onset of depression is well known (Fiske *et al*., 2009), however, to our knowledge the protective role of labour market participation has not yet been reported. It may be that young-old adults from the recent cohort have profited from changing societal attitudes towards being open about mental illness and seeking help, which may be related to higher average educational levels, increases in participation in gainful employment and higher experienced levels of mastery. Jorm *et al*. (2017) has found some evidence that improvements in treatment, particularly antidepressants, have been masked by an increased reporting of symptoms because of a greater public awareness of mental disorders or willingness to disclose (Jorm *et al*., 2017). This may be an explanation for the higher CES-D baseline score we found in the recent *v*. the early cohort.

The deteriorating health trends observed in our data are not surprising, and have previously been found in another study among older adults in the Netherlands (Van Oostrom *et al*., 2016). In western societies the overall prevalence of chronic diseases is increasing due to the ageing of the population and the greater longevity of people with chronic conditions (Crimmins and Beltrán-Sánchez, 2011). Also, mobility functioning has deteriorated and length of life with disease and mobility functioning loss has increased between 1998 and 2008. No major role for antidepressants was found in reducing the incidence of depression in the recent cohort at follow-up. This may be explained by the broad definition of depression used in this study (CES-D ⩾ 16), including both subthreshold and major depression. Since antidepressants are not the first-choice treatment for subthreshold depression, it could be that the potential of antidepressants in preventing a new episode of depression is underestimated in our study, when compared with studies investigating the incidence of major depression.

The most important strengths are the cohort-sequential study design in which identical methods were used to recruit and measure random samples of 55–64-year-olds in the Netherlands. We had large enough samples to be able to select those without depression at baseline, following them up for maximal 10 years testing for birth-cohort differences in the incidence of depression. The relatively rich collection of risk and protective factors has enabled systematically testing the effects of putative risk and protective explanatory factors. Because both cohorts are identically measured, using long-term follow-ups with low dropout rates, our finding is not likely the result of an artefact. The study focused on a broad, but well-defined, definition of incident depression (CES-D ⩾ 16), because it has become clear that mild depression has also a huge impact on the public health burden (Meeks *et al*., 2011).

A number of limitations must be acknowledged. The depression incidence may be underestimated since the occurrence of depression between three-yearly follow-up measurements could have been missed. Also, 3-year incidence rates might lead to a loss of power in Cox regression. However, this is not likely to have affected the cohort comparison, because each cohort had the same follow-up schedule. Furthermore, information on a depression history lacked for most respondents in both cohorts. Additionally, solely baseline explanatory factors were included to facilitate the interpretation of cohort differences found, which may be a less sensitive method than studying time-varying factors, as risk and protective factors can change during follow-up. About two-thirds of the decline in incidence could not be explained by factors included in this study, suggesting that other factors have been important also.

To conclude, this study has demonstrated that the depression incidence fluctuates, which is partly influenced by changing risk and protective factors of depression in the community. This also led to the identification of important targets for prevention strategies. For policy makers, the most important message is that the incidence of depression is far from a stable trait of a community, and that it can be influenced. As many of the important protective factors are essentially environmental and man-made factors, this shows that policies aiming to strengthen resilience can indeed have substantial effects on the incidence of a major mental illness, such as depression.

Future research should investigate whether the same trends can be found and explained in other age groups, and in other countries. Furthermore, also cohort differences in the natural course of depression have to be investigated, because the course together with the incidence of depression determines the prevalence, i.e. burden of disease. More detailed insight into time trends of depression will help the field to move forward in the global priority to reduce the disease burden of depression (Cuijpers *et al*., 2012).

## Data

An overview of the data used in this study can be found at https://www.lasa-vu.nl/data/availability_data/availability_data.htm. Data will not be shared, unless a specific request is made.
